# Morphometric Analysis of Subaxial Cervical Vertebra Pedicles in the Turkish Population

**DOI:** 10.3390/tomography11070079

**Published:** 2025-07-04

**Authors:** Hande Nur Taşdemir Batir, Hatice Güler, Burcu Kamaşak Arpaçay, İzzet Ökçesiz, Halil Dönmez, Güven Kahriman

**Affiliations:** 1 Department of Anatomy, Faculty of Medicine, Erciyes University, Kayseri 38280, Turkey; handenur.tsdmr@gmail.com; 2 Department of Anatomy, Faculty of Medicine, Kırşehir Ahi Evran University, Kırşehir 40080, Turkey; brc1608@hotmail.com; 3 Department of Radiology, Faculty of Medicine, Erciyes University, Kayseri 38280, Turkey; izzetokcesiz@erciyes.edu.tr (İ.Ö.); hdonmez@erciyes.edu.tr (H.D.); kahriman@erciyes.edu.tr (G.K.)

**Keywords:** cervical vertebra, morphometry, pedicle, screwing

## Abstract

Background/Objectives: One of the surgical interventions applied in the cervical region is the pedicle screw method. The cervical pedicle screw is stronger than any other screw method; however, use of the cervical pedicle screw is limited due to the variability in the anatomy of the cervical vertebrae and the risks to the neurological and vascular structures in this region. This study aimed to determine the morphological features of subaxial cervical vertebrae of the adult Turkish population and to provide guidance for the pedicle screwing method. Methods: In our study, pedicle analyses were examined in the subaxial neck vertebrae of a total of 60 patients, 30 male and 30 female, using computed tomography images. In subaxial vertebrae (C3–C7), bilateral pedicle width, pedicle axis length, pedicle transverse angle, sagittal and transverse diameter of vertebral foramen, and the distance between two pedicles were measured. Results: Pedicle widths that did not fit the commonly used 3.5 mm pedicle screw were detected in both male and female patients. The mean bilateral pedicle width in male patients was found to be greater than in female patients. When the parameter results were compared according to the levels, it was found that the pedicle width, pedicle axis length, transverse diameter, and the distance between the two pedicles increased statistically significantly. Conclusions: We think that the data obtained from the study will help determine the appropriate screwing (screw selection) in subaxial vertebra pedicle surgery and increase the success of the surgical procedure.

## 1. Introduction

**Figure 1:**
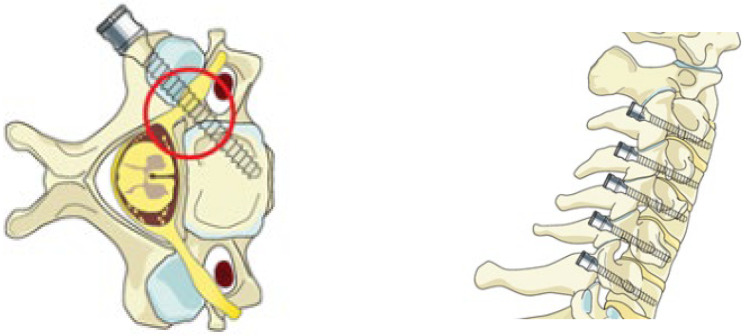
Pedicle screwing method in the cervical vertebrae [9].

The neck region is the most mobile part of the columna vertebralis, which is composed of 33 vertebrae arranged one on top of the other, and is an important part of the axial skeleton [1]. This situation is a factor that affects the movement mechanism, stability, and deterioration that may occur with trauma in the region [2,3]. In addition, the cervical vertebrae are an area frequently exposed to trauma and prone to various degenerative and neoplastic diseases. For such reasons, it requires frequent surgical intervention [4,5]. One of the surgical intervention methods performed in the region today is pedicle screwing. However, the widespread use of pedicle screws in surgery is limited due to the risk of injury to the medulla spinalis, spinal nerves and also the arteria vertebralis, caused by their close anatomical proximity [6,7,8], (Figure 1). 

Detailed anatomical knowledge of the cervical vertebrae is necessary for a successful surgery [4,5]. Among the morphometric measurements of the cervical vertebrae, pedicle measurements are useful data that surgeons use as a reference in pedicle screwing. Researchers believe that from a clinical practice perspective, these measurements are what are needed to understand pedicle morphology and plan appropriate surgical treatment. Measurements of the subaxial cervical spine and their use in the field of surgery have developed over the last 50 years. At the end of the 20th century, it was observed that lateral mass screw applications increased in surgical interventions in the cervical spine. Biomechanically, the cervical pedicle screw is much stronger than any lateral mass screw; however, widespread use of this technique has not yet been achieved in the neck region [10].

Pedicle screw insertion points and orientations have been found to differ significantly at most subaxial cervical vertebrae levels and between genders [7,11]. Ethnic variability in cervical pedicle anatomy has been demonstrated in previous studies [10]. Since the variability in vertebra sizes between different enthicities prevents the standardization of measurements, the necessity of measurements according to ethnicity arises [4,5].

The paucity of studies on the morphology of cervical vertebrae pedicles in the literature is striking. With this study, we aim to increase the database for pedicle measurements in the Turkish population, to expand the database for correct and healthy application in surgery, and to quantify the data in our target population and compare it with the existing data obtained from other studies.

## 2. Materials and Methods

Computed Tomography (CT) images used in the study belong to patients who applied to the Erciyes University Faculty of Medicine Radiology Polyclinic. The images taken in DICOM format were transferred to the Microdicom program and measurements were made. In the study, the pedicle analyses of the subaxial neck vertebrae of a total of 60 patients, 30 female and 30 male between the ages of 18–45, were examined. Patients with any congenital anomalies, surgical procedures, neoplastic lesions, and trauma in the cervical region were not included in the study.

Statistical power analysis of the study was performed (accepting Alpha: 0.05, power (1-Beta): 0.80 and effect size: 0.8) and a total of 52 patients were calculated, with at least 26 patients for each group. We screened nearly 2000 patients who applied to Erciyes University Faculty of Medicine Radiology Polyclinic. In order to achieve healthy results, we determined patients over the age of 18 in terms of bone development completion and under the age of 45 in terms of excluding age-related osteoporosis. We examined our other exclusion criteria one by one and could only reach 60 patients in total.

Cervical region images taken with the multi-slice CT device (MSCT) (General Electric IQTM 32-Detector Spiral MSCT) without contrast material while the individuals were in anatomical position were evaluated. The acquisition parameters were set as 120 kV, WL: 350, WW: 2000 and 5 mm slice thickness. The images taken in the axial section were evaluated.

For measurements, CT images with a slice thickness of 5 mm were recorded in DICOM format. All these images were viewed simultaneously in the “microdicom” program, which can be downloaded from https://www.microdicom.com/downloads.html (accessed on 11 March 2022). The length and angle of any specific point (or area) on the image were measured using the options in the “measurement and tools” tab in the Microdicom program. The entire procedure was repeated bilaterally on the CTs for the right and left cervical vertebrae of each participant. In order to avoid bias, measurements were made by a single anatomist with approximately ten years of experience. Measurements made by a single person are also applied in the literature and do not cause any limitations [12].

**Figure 2:**
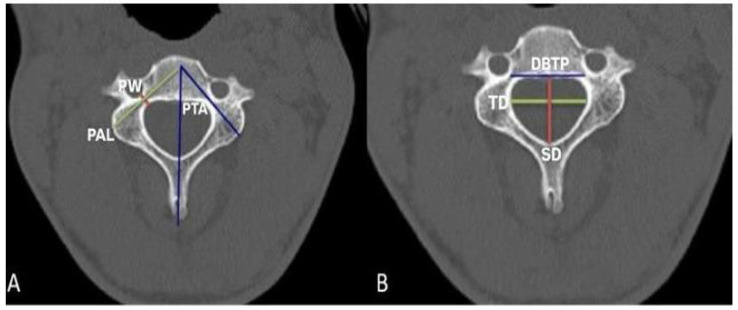
View of pedicle (**A**): PW: Pedicle width, PTA: Pedicle transverse angle, PAL: Pedicle axis length. (**B**): SD: Sagittal diameter, TD: Transverse diameter, DBTP: Distance between two pedicles.

Measurements performed (C3–C7) pedicle width (PW), pedicle axis length (PAL), pedicle transverse angle (PTA), sagittal diameter (SD) and transverse diameter (TD) of the vertebral foramen, and the distance between two pedicles (DBTP) were measured (Figure 1). Pedicle width was measured bilaterally as pedicle width right (PWR) and pedicle width left (PWL). Pedicle axis length was measured bilaterally as pedicle axis length right (PALR) and pedicle axis length left (PALL). Pedicle transverse angle was measured bilaterally as pedicle transverse angle right (PTAR) and pedicle transverse angle left (PTAL). The measurements are shown in Figure 2.

Pedicle width measurement is taken in mm from the point where the pedicle is thinnest in the medio-lateral direction in the widest section of the vertebral pedicle. Pedicle axis length measurement is the length of the vertebral pedicle axis in mm, starting from the posterior of the vertebra and extending to the sagittal plane that divides the vertebra into two equal parts, right and left. Pedicle transverse angle measurement is in degrees that the vertebral pedicle makes with the sagittal plane. Sagittal diameter measurement is the widest diameter of the vertebral foramen in the antero-posterior direction in mm. Transverse diameter measurement is the widest diameter of the vertebral foramen in the medio-lateral direction in the transverse plane in mm. The distance between the two pedicles is the distance between the points where the pedicle widths of the vertebrae and the pedicle axis lengths intersect, in mm.

### Statistical Analysis

Statistical analysis was performed using SPSS 22.0 program. The average values, standard deviations and correlations of the measurements taken were examined. Independent two sample *t*-test and Pearson correlation analysis were used in SPSS program. The measurement results were interpreted as *p* < 0.001 as very high statistical significance, 0.001 ≤ *p* < 0.01 as high statistical significance, 0.01 ≤ *p* < 0.05 as statistically significant, 0.05 ≤ *p* < 0.10 as borderline significance, and *p* > 0.10 as no statistically significant difference.

## 3. Results

Pedicle widths, pedicle transverse angles, pedicle axis lengths right and left, as well as the transverse diameter and sagittal diameter of the foramen vertabralis, and the distance between two pedicles are given in Table 1 with their standard deviations and average values for male, female, and general.

Table 1 shows statistical comparisons of PWR, PWL, PTAR, PTAL, PALR, PALL, TD, SD, and DBTP measurements for each vertebral level among males and females aged 18–45 years. The “*p*” value is indicated below each measurement. The +, − values indicate the maximum deviation of individual values from the average.

When male and female patients were compared, the average right and left pedicle widths in all vertebrae were found to be greater in male than in female. In both male and female, the mean values of the right and left pedicle width were found to be close to each other within their own gender. In both genders, the lowest pedicle width was C3 on the right and left sides, while the highest average belonged to the C7 vertebra. Pedicle width average from C3 to C7 was observed on the right and left sides in both genders.

The average right and left pedicle transverse angles were found to be greater in male than in female. The mean values of the right and left pedicle transverse angles of the vertebrae in both male and female were found to be close to each other. The lowest angle averages on the right and left sides for both genders were found in the C7 vertebra. The highest angle average in male was found in C4, while the highest angle average in female was found in the C5 vertebra.

The mean right and left pedicle axis lengths in all vertebrae were found to be greater in male than in female. In male and female, the mean values of pedicle axis length measurements on the right and left sides of all vertebrae were found to be close to each other. The average pedicle axis length on the right and left sides was lowest in the C3 vertebra in male, while it was found in the C4 vertebra in female. The highest value was found in the C7 vertebra in both genders.

The mean transverse diameters were found to be larger in male than in female in all vertebrae. The lowest mean transverse diameter in males and females belonged to the C3 vertebra. The highest mean transverse diameter was found in C7 vertebra in male and C6 vertebra in female.

The mean sagittal diameters were found to be larger in male than in female in all vertebrae. The lowest mean sagittal diameter in both genders belonged to the C4 vertebra. The highest mean sagittal diameter was found in C7 vertebra in male and C6 vertebra in female.

The average distance between two pedicles was found to be greater in male than in female in all vertebrae. In males and females, the distance between two pedicles was highest in the C7 vertebra and lowest in the C3 vertebra. The average distance between two pedicles increased from C3 to C7 in male, female, and the general population.

## 4. Discussion

Cervical vertebrae are exposed to serious injuries, instability, and degenerative changes due to their different kinesiology and anatomical structure. Therefore, they require frequent surgical intervention [4]. The risk posed by the neurological and vascular structures in this region has limited the widespread use of pedicle screws [7,10]. Although the cervical pedicle screw, which is biomechanically much stronger than any lateral mass screw, is the safest form of fixation for surgeons, its widespread use has not yet been achieved due to its feasibility in the cervical region [10]. Since ethnic variations in cervical pedicle anatomy, gender-related differences, and variations in cervical vertebral levels prevent standardization of measurements, measurement according to ethnicity is necessary [4,7]. Accordingly, the pedicle width, pedicle transverse angle, pedicle axis length, transverse diameter, and sagittal diameter measurements obtained from our study are given in Table 2, comparatively by ethnicity.

One of the most important data in pedicle screwing is pedicle width. Rao et al. (2008) [7] reported in their study that the average pedicle width at all subaxial levels was sufficient for screws with a diameter of 3.5 mm (in 98% of the volunteers). Although the prevalence is low, they stated that female patients are more likely to have pedicle width dimensions of less than 4.0 mm. Similarly, our data show that pedicle width in female patients may be less than 4.0 mm more frequently than in male patients.

Patwardhan et al. (2012) [14] emphasized that the pedicle transverse diameter of both male and female patients was less than 5.0 mm in some samples, so 3.5 mm pedicle screws could not be used in fixation. They commented that for the Indian population, 2.7 mm screws could be designed to give a wider margin of safety. When the mean pedicle width measurements in our study were compared with the Indian population in this study, all mean pedicle width measurements except C7 in males were found to be lower in our study compared to the Indian population (Table 2).

Munusamy et al. (2015) [15] reported in their study that the minimum mean pedicle width was at C4 in males and C3 in females. They reported that they rarely encountered a pedicle of a width less than 4.00 mm in their study groups. In our study, similarly, the mean pedicle width was found to be greater in males than in females at all levels; however, the smallest mean pedicle width was found to be C3 for both genders (*p* < 0.05). In our study, we encountered a certain number of pedicle widths less than 4.0 mm at each level, especially in female, from C3 to C7. This shows that ethnic differences have an important effect on the reliability of pedicle screwing.

Canberk (2017) [16] found the smallest average width in the lower cervical vertebrae in the pedicle of the C3 vertebra. He determined that the thickest pedicle width was that of the C7 vertebra and that it was the most suitable cervical vertebra for screw fixation. Our study further supports this study by showing that pedicle width is smaller in the Turkish population compared to other ethnic groups.

Farooque et al. (2018) [17] confirmed in their study that cervical pedicle screw placement is possible in most of the Indian population except C3 in female. According to our study, pedicle widths below 3.00 mm were rarely measured at C3, C4, C5, and C6 in female. Therefore, levels other than C3 should be carefully analyzed with preoperative CT.

Westermann et al. (2018) [18] found the average pedicle width to be smaller in female patients than in male patients. They reported that more than half of the female patients and almost one-third of the male patients had a pedicle width of less than 4.5 mm at the C3 level. Although this decreases caudally, they reported that it would not be safe to place pedicle screws in a significant percentage of patients. In our study, we determined that almost all females and almost half of males had a pedicle width of less than 4.5 mm at the C3 level. This situation generally decreases towards the caudal direction in our study as well. Our study shows that the Turkish population is at higher risk for pedicle screwing according to this study.

Alsaleh et al. (2021) [10] stated that the smallest pedicle width in the general population is 4.26 mm at C3. In our study, we determined that the smallest pedicle width in the Turkish population is C5 and is 2.62 mm.

According to our study, preoperative CT evaluation is clearly a necessity before transpedicular fixation in the cervical spine, especially in the Turkish female population in subaxial cervical vertebrae. In female patients, 22.3% of the pedicle width measurements were between 3 mm and 4 mm, while 3.6% were less than 3 mm. In male patients, 6.3% of the pedicle width measurements were between 3 mm and 4 mm. Pedicle widths below 3.0 mm, which are rarely seen at any level except C7, make the use of 3.5 mm pedicle screws, which are commonly used in these individuals, not possible. Therefore, detailed CT analysis in the Turkish population shows that the use of alternative screwing or fixation methods in the subaxial cervical spine or the production of smaller diameter pedicle screws is mandatory for the reliability of surgery.

Abumi et al. (1994) [13], who first performed pedicle screw measurements in 1994, applied cervical pedicle screw to 13 patients with spinal damage in the cervical region for treatment purposes. As a result of their study, the pedicle transverse angle was found to be less than in our study.

Rao et al. (2008) reported in their study that the lowest average pedicle transverse angle was at C7 in females and the highest at C4 in males (Table 2). In our study, we also determined that the lowest average pedicle transverse angle was at C7 and the highest at C4 in both male and female patients [7].

Munusamy et al. (2015) reported in their study that the lowest mean pedicle transverse angle was at C7 in females and the highest was at C4 in females (Table 2). The values for the average pedicle transverse angle in our study have similar ranges to this study [15].

Canberk (2017) determined the average pedicle transverse angle of individuals between the ages of 18–96 to be the lowest at C7 in females and the highest at C4 in females (Table 2). We included individuals between the ages of 18–45 in our study, so we think that the difference between Canberk’s study and our study may be due to the age range of the participants [16].

Farooque et al. (2018) reported the mean pedicle transverse angle as the lowest at C7 in females and the highest at C3 in males in their study. Westermann et al. (2018) reported the mean pedicle transverse angle as the lowest at C7 in females and the highest at C4 in males in their study [17]. Atalar (2018) determined the mean pedicle transverse angle to be the lowest at C7 in females and the highest at C4 in males [19]. Alsaleh (2021) reported the mean pedicle transverse angle to be the lowest at C7 and the highest at C5 in his study [10] (Table 2). In our study, we determined the mean pedicle transverse angle for subaxial cervical vertebrae to be the lowest at C7 in females and the highest at C4 in males.

Rao et al. (2008) found the mean pedicle axis length to be the highest at C4 in males and the lowest at C7 in females in their study [7]. Farooque et al. (2018) reported the mean pedicle axis length to be the highest at C7 in males and the lowest at C3 in females in their study [17]. Westermann et al. (2018) reported the mean pedicle axis length to be the highest at C6 in males and the lowest at C4 in females in their study [18]. Atalar (2018) found the highest mean pedicle axis length at C4 in females and the lowest at C7 in males [19]. In our study, we found the highest mean pedicle axis length at C7 in males and the lowest at C4 in females. Similar ranges of measurement values were obtained with previous studies [7,17,18] and smaller values were found in females than in males. In addition, mean pedicle axis lengths close to each other were determined at each level. We think that different values were obtained in the study of Atalar (2018) due to the use of different measurement methods [19].

The values found for transverse diameter and sagittal diameter in Canberk’s (2017) study are similar to our study [16] (Table 2).

When our study is compared with other studies, although it shows similar characteristics in terms of pedicle transverse angle and pedicle axis length values, smaller values were obtained in terms of pedicle width. This is also very important in terms of surgery. Therefore, in the Turkish population, the cervical region should be evaluated and planned meticulously before surgery. In addition, in order to minimize the risks to the surrounding structures of the vertebra during pedicle screwing in cervical vertebrae, it is important to include other structures of the vertebra in the measurements in addition to the pedicle measurements.

When compared with different ethnicities, the pedicle axis length and pedicle transverse angle in the Turkish population showed similar characteristics, while the pedicle width was found to be smaller than in other ethnic groups. In the Turkish population, especially in females, pedicle widths of less than 3 mm were detected in some vertebral pedicles from C3 to C6. Accordingly, the use of 3.5 mm pedicle screws, which are commonly used in these vertebral pedicles, is restricted. Therefore, the appropriate screw diameter or alternative fixation methods should be determined in these vertebrae. Apart from this, cervical pedicle screwing is suitable for other individuals after detailed analysis with preoperative CT scan specific to each individual.

With this study, the database has been expanded in addition to existing studies for subaxial cervical vertebrae belonging to the Turkish population. We believe that the measurements will be a reference for preoperative planning and determining the appropriate pedicle screw width. In addition to this, due to the risks in the cervical region, measurements of the transverse diameter, sagittal diameter and the distance between the two pedicles will facilitate the identification of structures around the pedicle. Therefore, we think that these measurements will reduce the risk in the region and make the surgery more reliable.

## Figures and Tables

**Table 1:** Results of pedicle measurements according to vertebra levels and gender.

Levels	G	PWR	PWL	PTAR	PTAL	PALR	PALL	TD	SD	DBTP
C3	M	4.76 ± 0.72	4.61 ± 0.70	48.35 ± 5.31	47.71 ± 3.48	30.74 ± 2.14	30.86 ± 1.94	24.63 ± 1.44	15.10 ± 1.76	25.85 ± 1.10
	F	4.11 ± 0.63	3.91 ± 0.58	46.40 ± 4.11	47.08 ± 3.59	29.70 ± 2.16	27.79 ± 1.49	22.73 ± 1.19	13.99 ± 1.48	24.13 ± 0.84
	GN	4.44 ± 0.74	4.26 ± 0.73	47.38 ± 4.81	47.39 ± 3.52	30.22 ± 2.19	29.83 ± 20.01	23.68 ± 1.62	14.55 ± 1.70	24.99 ± 1.30
	*p*	0.001	<0.001	0.117	0.491	0.065	<0.001	<0.001	0.010	<0.001
C4	M	4.85 ± 0.71	4.80 ± 0.80	50.61 ± 4.42	50.81 ± 3.69	30.78 ± 1.96	30.88 ± 2.05	25.79 ± 1.33	14.19 ± 1.50	26.69 ± 1.26
	F	4.29 ± 0.80	4.13 ± 0.73	48.74 ± 3.99	49.65 ± 3.37	29.35 ± 2.09	24.79 ± 1.33	24.25 ± 1.19	13.55 ± 1.63	25.26 ± 1.25
	GN	4.57 ± 0.80	4.46 ± 0.83	49.68 ± 4.28	50.23 ± 3.55	30.06 ± 2.13	29.80 ± 2.07	25.02 ± 1.47	13.87 ± 1.58	25.97 ± 1.44
	*p*	0.005	0.001	0.091	0.209	0.009	<0.001	<0.001	0.115	<0.001
C5	M	5.25 ± 0.77	5.18 ± 0.77	49.04 ± 3.96	50.31 ± 4.14	32.54 ± 1.90	32.49 ± 1.95	27.25 ± 1.79	14.98 ± 1.69	27.84 ± 1.35
	F	4.61 ± 0.67	4.44 ± 0.72	48.93 ± 4.32	50.60 ± 4.25	30.31 ± 1.95	29.29 ± 1.62	25.39 ± 1.57	13.73 ± 1.72	25.82 ± 1.16
	GN	4.93 ± 0.78	4.81 ± 0.83	48.99 ± 4.11	50.45 ± 4.16	31.42 ± 2.21	30.89 ± 2.40	26.32 ± 1.91	14.36 ± 1.81	26.83 ± 1.61
	*p*	0.001	<0.001	0.920	0.794	<0.001	<0.001	<0.001	0.006	<0.001
C6	M	5.59 ± 0.77	5.36 ± 0.73	46.77 ± 5.53	46.38 ± 5.30	33.78 ± 2.39	33.52 ± 2.54	27.54 ± 1.83	15.96 ± 2.00	29.24 ± 1.77
	F	4.65 ± 0.90	4.50 ± 0.65	44.24 ± 4.60	45.88 ± 3.64	31.24 ± 2.25	30.77 ± 2.41	25.55 ± 1.77	14.47 ± 1.86	26.69 ± 1.46
	GN	5.12 ± 0.96	4.93 ± 0.81	45.01 ± 5.10	46.13 ± 4.51	32.51 ± 2.63	32.15 ± 2.82	26.54 ± 2.05	15.21 ± 2.06	27.97 ± 2.06
	*p*	<0.001	<0.001	0.251	0.671	<0.001	<0.001	<0.001	0.004	<0.001
C7	M	6.31 ± 10.89	6.22 ± 0.93	40.74 ± 4.56	41.29 ± 5.37	35.35 ± 3.03	34.92 ± 2.72	2.85 ± 1.90	16.65 ± 2.35	30.35 ± 1.60
	F	5.39 ± 0.83	5.45 ± 0.65	34.97 ± 4.37	38.74 ± 2.91	31.97 ± 3.34	31.66 ± 3.13	24.65 ± 1.81	14.37 ± 1.87	28.44 ± 1.35
	GN	5.85 ± 0.97	5.84 ± 0.89	37.85 ± 5.30	40.02 ± 4.47	33.66 ± 3.59	33.83 ± 3.35	25.75 ± 2.15	15.51 ± 2.40	29.39 ± 1.76
	*p*	<0.001	<0.001	<0.001	0.025	<0.001	<0.001	<0.001	<0.001	<0.001

(G: Gender, F: Female, M: Male, GN: General, PWR: Pedicle Width Right, PWL: Pedicle Width Left, PTAR: Pedicle Transverse Angle Right, PTAL: Pedicle Transverse Angle Left, PALR: Pedicle Axis Length Right, PALL: Pedicle Axis Length Left, TD: Transverse Diameter, SD: Sagittal Diameter, DBTP: Distance Between Two Pedicles).

**Table 2:** Comparison of morphometric measurements made on subaxial cervical vertebrae (Studies in the literature between 1994–2023).

	ETHNICITY	AGE/ GENDER	PW	PTA	PAL	TD	SD
ABUMI, 1994 [13]		15–80/ 12M-1F		30–40			
RAO, 2008 [7]	North American	average 25/ 63M-35F	M-F C3 5.8–4.8 C4 6.0–5.0 C5 6.3–5.2 C6 6.5–5.7 C7 7.6–6.5	M-F C3 47.4–46.6 C4 47.8–47.8 C5 45.9–46.9 C6 41.8–42.5 C7 33.8–33.0	M-F C3 34.3–30.9 C4 33.7–30.3 C5 34.2–30.9 C6 34.1–30.6 C7 32.6–28.9		
PATWARDHAN, 2012 [14]	Indian	-/27M,F	M-F C3 5.3–4.6 C4 5.3–4.7 C5 5.6–4.7 C6 5.6–5.3 C7 6.1–5.6				
MUNUSAMY, 2015 [15]	Multiracial Asia (33 Chinese, 11 Malay, 6 Indian)	average 38.5 (21–70)/ 31M-19F	M-F C3 5.74–4.75 C4 5.70–4.77 C5 6.07–5.18 C6 6.42–5.45 C7 7.07–6.29	M-F C3 45.7–46.7 C4 48.4–48.8 C5 47.9–47.6 C6 44.0–44.9 C7 38.2–37.8			
CANBERK, 2017 [16]	Turkish	18–96/ 54M-46F	RIGHT M-F C3 4.22–3.58 C4 4.11–3.55 C5 4.91–3.92 C6 5.26–4.70 C7 6.03–5.10 LEFT M-F C3 4.31–3.35 C4 4.40–3.39 C5 4.77–4.34 C6 5.37–4.65 C7 5.99–4.75	RIGHT M-F C3 36.14–39.25 C4 37.74–38.94 C5 35.34–36.31 C6 33.36–33.73 C7 30.99–30.57 LEFT M-F C3 39.44–38.46 C4 41.68–43.63 C5 38.87–38.89 C6 38.91–40.59 C7 33.29–33.51		M-F C3 23.98–23.06 C4 25.02–24.41 C5 25.56–25.29 C6 26.18–25.35 C7 25.50–23.60	M-F C3 15.23–12.78 C4 14.40–12.47 C5 14.76–13.05 C6 15.12–14.19 C7 15.02–14.03
FAROOQUE, 2018 [17]	Indian	E-Average 29.3 F-Average 31.3/ 50M-50F	RIGHT M-F C3 4.72–4.37 C4 5.00–4.60 C5 5.49–4.83 C6 5.79–5.00 C7 6.10–5.47 LEFT M-F C3 4.74–4.30 C4 5.08–4.61 C5 5.44–4.80 C6 5.70–5.08 C7 6.00–5.42	RIGHT M-F C3 45.01–44.02 C4 43.40–41.92 C5 41.60–40.26 C6 39.80–38.42 C7 37.72–36.58 LEFT M-F C3 44.94–44.00 C4 43.80–41.94 C5 41.55–40.02 C6 39.20–38.28 C7 37.63–36.64	RIGHT M-F C3 30.29–28.89 C4 30.76–29.46 C5 31.52–30.41 C6 32.61–31.78 C7 34.23–32.33 LEFT M-F C3 30.32–28.90 C4 30.84–29.59 C5 31.67–30.60 C6 32.41–31.81 C7 33.19–32.43		
WESTERMANN, 2018 [18]	Caucasian	E-Average 58 F-Average 57/ 52M-48F	RIGHT M-F C3 4.99–4.34 C4 5.27–4.38 C5 5.50–4.84 C6 6.12–5.16 C7 6.73–6.00 LEFT M-F C3 4.88–4.08 C4 5.13–4.38 C5 5.59–5.03 C6 5.99–5.18 C7 6.87–6.10	RIGHT M-F C3 44.54–45.27 C4 48.37–47.64 C5 47.38–47.34 C6 43.55–43.46 C7 35.72–35.43 LEFT M-F C3 44.72–45.26 C4 47.95–47.21 C5 47.41–45.90 C6 43.36–41.77 C7 34.79–34.40	RIGHT M-F C3 32.47–30.51 C4 31.76–29.72 C5 33.45–31.05 C6 35.26–31.13 C7 34.74–31.17 LEFT M-F C3 32.41–30.07 C4 31.69–29.20 C5 32.72–30.62 C6 34.48–31.31 C7 34.49–30.72		
ATALAR, 2018 [19]	Turkish	18–30, 31–50, 51+/ 41M-59F		RIGHT M-F C3 40.80–42.00 C4 44.83–44.90 C5 44.62–42.06 C6 38.13–36.72 C7 28.57–27.40 LEFT M-F C3 43.00–42.87 C4 46.55–44.72 C5 44.45–44.42 C6 39.46–38.31 C7 29.37–30.01	RIGHT M-F C3 17.8–25.4 C4 17.8–25.7 C5 18.9–25.2 C6 19.1–22.4 C7 17.0–19.9 LEFT M-F C3 17.6–26.2 C4 17.12–27 C5 17.6–25.2 C6 18.2–24.6 C7 17.4–22.6		
ALSALEH, 2021 [10]	Middle East	average 40 (18–92)/ 154M-116F	C3 4.4 C4 4.4 C5 4.8 C6 5 C7 6.1	C3 44.1 C4 46.7 C5 47.3 C6 44.2 C7 35.5			
PRESENT STUDY, 2023	Turkish	18–45/ 30M-30F	RIGHT M-F C3 4.76–4.11 C4 4.85–4.29 C5 5.25–4.61 C6 5.59–4.65 C7 6.31–5.39 LEFT M-F C3 4.61–3.91 C4 4.80–4.13 C5 5.18–4.44 C6 5.36–4.50 C7 6.22–5.45	RIGHT M-F C3 48.35–46.40 C4 50.61–48.74 C5 49.04–48.93 C6 45.77–44.24 C7 40.74–34.98 LEFT M-F C3 47.71–47.08 C4 50.81–49.65 C5 50.31–50.60 C6 46.38–45.88 C7 41.29–38.73	RIGHT M-F C3 30.74–29.70 C4 30.78–29.35 C5 32.54–30.31 C6 33.78–31.25 C7 35.35–31.98 LEFT M-F C3 30.86–28.79 C4 30.88–28.71 C5 32.49–29.29 C6 33.52–30.78 C7 34.94–31.66	M-F C3 24.63–22.73 C4 25.79–24.25 C5 27.25–25.39 C6 27.54–25.55 C7 28.85–24.65	M-F C3 15.10–13.99 C4 14.19–13.55 C5 14.98–13.73 C6 15.96–14.47 C7 16.65–14.37

(F: Female, M: Male, PW: Pedicle Width, PTA: Pedicle Transverse Angle, PAL: Pedicle Axis Length, TD: Transverse Diameter, SD: Sagittal Diameter).

## Data Availability

The data that support the findings of this study are available on request from the corresponding author.
